# The roles of herbal remedies in survival and quality of life among long-term breast cancer survivors - results of a prospective study

**DOI:** 10.1186/1471-2407-11-222

**Published:** 2011-06-06

**Authors:** Huiyan Ma, Catherine L Carpenter, Jane Sullivan-Halley, Leslie Bernstein

**Affiliations:** 1 Department of Population Sciences, Beckman Research Institute, City of Hope, Duarte, CA, USA; 2 Department of Medicine, David Geffen School of Medicine at UCLA, Los Angeles, CA, USA

**Keywords:** herb, breast cancer, survival, mortality, QOL

## Abstract

**Background:**

Few data exist on survival or health-related quality of life (QOL) related to herbal remedy use among long-term breast cancer survivors. The objective of this report is to examine whether herbal remedy use is associated with survival or the health-related QOL of these long-term breast cancer survivors.

**Methods:**

In 1999-2000, we collected the information of herbal remedy use and QOL during a telephone interview with 371 Los Angeles Non-Hispanic/Hispanic white women who had survived more than 10 years after breast cancer diagnosis. QOL was measured using the Medical Outcomes Study Short Form-36 (SF-36) questionnaire. Patients were followed for mortality from the baseline interview through 2007. 299 surviving patients completed a second telephone interview on QOL in 2002-2004. We used multivariable Cox proportional hazards methods to estimate relative risks (RR) and 95% confidence intervals (CI) for mortality and applied multivariable linear regression models to compare average SF-36 change scores (follow-up - baseline) between herbal remedy users and non-users.

**Results:**

Fifty-nine percent of participants were herbal remedy users at baseline. The most commonly used herbal remedies were echinacea, herbal teas, and ginko biloba. Herbal remedy use was associated with non-statistically significant increases in the risks for all-cause (44 deaths, RR = 1.28, 95% CI = 0.62-2.64) and breast cancer (33 deaths, RR = 1.78, 95% CI = 0.72-4.40) mortality. Both herbal remedy users' and non-users' mental component summary scores on the SF-36 increased similarly from the first survey to the second survey (*P *= 0.16), but herbal remedy users' physical component summary scores decreased more than those of non-users (-5.7 vs. -3.2, *P *= 0.02).

**Conclusions:**

Our data provide some evidence that herbal remedy use is associated with poorer survival and a poorer physical component score for health-related QOL among women who have survived breast cancer for at least 10 years. These conclusions are based on exploratory analyses of data from a prospective study using two-sided statistical tests with no correction for multiple testing and are limited by few deaths for mortality analysis and lack of information on when herbal remedy use was initiated or duration of or reasons for use.

## Background

While the use of complementary and alternative medicine (CAM, including herbal remedies), has increased among the general population [1]. CAM use still remains more common among cancer patients [2]. Among cancer patients, breast cancer patients remain as the most likely users of CAM [3]. The prevalence of CAM among cancer patients varies based on different CAM definitions [4,5]. Richardson et al. reported that as many as 83% breast cancer patients use at least one type of CAM [6]. The leading reasons why breast cancer patients use CAM include improving their quality of life (QOL), aiding their conventional medical treatment for cancers, preventing their cancer recurrence, and eventually extending their lifespan [7,8]. However, few scientific data exist on survival or QOL in relation to CAM use, especially herbal remedy use, among long-term breast cancer survivors.

The relationship between CAM or herbal remedy use and cancer patients' survival has rarely been studied. One Norwegian study following 252 patients with a mix of different cancers for 5 years reported that CAM use had no impact on mortality [9]. Another Norwegian study following 515 patients with different types of cancer for 8 years found that CAM users had a 30% higher mortality risk (*P *= 0.056) during the follow-up period than non-CAM users [10]. Neither study provided data specifically for breast cancer patients.

Most previous studies that evaluated CAM or herbal remedy use by patients in relation to QOL were conducted within 5 years of their initial diagnosis [11-14]. Recently, long-term breast cancer survivors' QOL has started to attract attention from researchers [15-17]. Such research is needed, especially given that early detection and efficacious adjuvant systemic therapy of breast cancer have improved the likelihood of long-term survival. Based on the data from the National Cancer Institute's Surveillance, Epidemiology, and End Results (SEER) Program, the proportion of patients with stage I (T1a,bN0M0) breast cancer diagnosed between 1988 and 2001 who remain alive at 10 years post-diagnosis is 76% [18]. Younger women who become long-term survivors commonly experience late health effects of treatment, symptoms associated with a deficit of estrogen, fear of recurrence, problems with sexuality, and poorer mental health and psychologic distress [15,17]. In a survey on beliefs about the roles of CAM use conducted among long-term breast cancer survivors (on average, 8.7 year post-diagnosis), more than 50% believed that CAM use would prevent cancer recurrence (69%), play a more active role in recovery (67%), and help to manage stress (64%) [19] although thus far, few scientific data exist to support these beliefs.

We previously reported that, among women who were long-term (> 10 years) breast cancer survivors and aged 40 years or younger when initially diagnosed, herbal remedy users had a lower mental component summary score on the Medical Outcomes Study Short Form-36 (SF-36) questionnaire than non-users, but herbal remedy users and non-users did not differ on the SF-36 physical component summary scores [20]. In our previous report, QOL and herbal remedy use information were collected at the same point in time and we were thus not able to determine whether herbal remedy use is associated with later QOL. However, additional follow-up data on these patients' QOL collected 2-5 years after the initial collection of herbal information now affords us the opportunity to evaluate whether herbal remedy use at baseline is associated with the changes in SF-36 QOL measures between the two surveys. Furthermore, the extended follow-up data on women's survival from baseline (1999-2000) through 2007 provides the opportunity to explore whether herbal remedy use at baseline is associated with mortality among long-term breast cancer survivors. In this report, we examined whether herbal remedy use is associated with survival or the health-related QOL of these long-term breast cancer survivors.

## Methods

### Study population

Participants were breast cancer patients enrolled in a Los Angeles County population-based epidemiologic case-control study of breast cancer among Non-Hispanic/Hispanic white women who were 40 years or younger at diagnosis (referred to as the "parent study"). Details of the parent study have been published [21]. Briefly, white female residents of Los Angeles County diagnosed with first primary *in situ *or invasive breast cancer between July 1, 1983, and January 1, 1989, who were 40 years of age or younger at diagnosis, and who were born in the United States, Canada, or Europe, were eligible for participation in the parent study. Nine hundred and sixty nine eligible breast cancer patients were identified by the Los Angeles County Cancer Surveillance Program (LA-CSP), a population-based SEER registry. Of the 969 eligible women with incident breast cancer, 225 were not interviewed for the following reasons: death (n = 20), physician refusal (n = 54), serious mental or physical illness (n = 7), patient refusal (n = 111), no longer living in Los Angeles County (n = 12), and inability to be located (n = 21). Interviews were completed with 744 breast cancer patients (*in situ*: n = 68, invasive: n = 676).

The 744 participants were traced in 1998 and interviewed in 1999 and 2000 by telephone to collect the detailed information regarding herbal remedy use and concurrent QOL. Of the 744 women, 276 were known to have died by the time the baseline telephone interview for herbal remedy use was initiated; 78 women were not located; 6 subjects refused participation; and 10 women were interested but unable to schedule an interview during the study period. Three hundred seventy-four women were interviewed. The interviews were conducted, on average, 13 years after diagnosis. The cross-sectional relationship between herbal remedy use and QOL has been reported elsewhere [20].

The 374 women who participated in our baseline telephone interview for herbal remedy use were followed for survival through December 31, 2007 by linking with the LA-CSP and the National Death Index. If death occurred, information on date of death and cause of death was collected. Person-days of follow-up for each woman began on the date of baseline telephone interview and ended on her date of death (n = 44 including 33 who died of breast cancer) or December 31, 2007 (n = 330). Among 33 who died from breast cancer, 13 had metastasis of breast cancer; 2 had recurrence of breast cancer; 2 had a contralateral breast cancer; 10 had a combination of these three conditions, and the other 6 women did not have any of these three conditions by the time of our baseline telephone interview. We excluded three women with incomplete questionnaire data who were alive at the end of the follow-up period. Therefore, 371 women were eligible for survival analysis.

**Figure 1:**
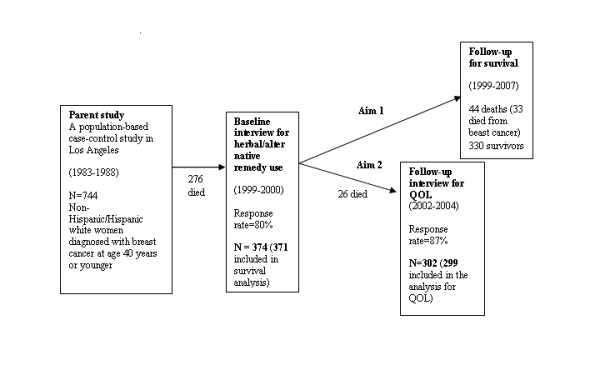
**Participant recruitment**.

We conducted a follow-up telephone interview in 2002-2004 to collect additional SF-36 QOL information 2-5 years after the initial collection of herbal remedy and QOL information. Of 374 case patients participating in the baseline telephone interview, 26 were known to have died when we began the follow-up interview. Of the remaining 348 women, 17 women were not located (4.9%); 26 subjects preferred not to participate (7.5%); and 3 women were too ill to participate (0.9%). Three hundred two women were interviewed. After excluding the same three women with incomplete questionnaire data, 299 women remained for analysis of the association between herbal remedy use and QOL. The recruitment results are combined and shown in Figure 1.

### Data collection

In the parent study, we obtained data on summary stage at breast cancer diagnosis from the LA-CSP. The baseline telephone interview for herbal remedy use (1999-2000) obtained information on herbal remedy use, SF-36 QOL measures, psychosocial functioning, cancer-related factors including recurrences, breast-cancer surgeries and reconstructive history, treatments received for breast cancer, and comorbidities. We also updated demographic information and information on lifestyle practices after breast cancer diagnosis.

Women were given a list of herbal remedies and asked whether they had used any of the listed remedies within the past six months. The remedies listed were bee pollen, licorice root, black cohosh, mother wort, blue cohosh, nux vomica, chaste berries, progesterone topical (vitex agnus cactii), progesterone cream (wild Mexican yam), chickweed tincture, pulsatilla, dong quai (tong Kwai), royal jelly, echinacea, sage tea, evening primrose oil, sarsaparilla, false unicorn, sepia, garlic, St. John's Wort, ginko biloba, valeriana, ginseng, wild yam root, herbal tea used as a remedy, shark cartilage, or lachesis. Use of any of the herbal remedies qualified a woman as an herbal remedy user.

The follow-up telephone interview (2002-2004) again measured SF-36 QOL and psychosocial functioning; updated demographic information and lifestyle practices and cancer-related and treatment-related factors including additional surgeries. Subjects were mailed a list of scale responses to the QOL and psychosocial instruments prior to each of their telephone interviews to facilitate the interview process.

Health-related QOL was assessed using the SF-36 questionnaire at each telephone interview. This widely used self-report measure contains 36 items in eight subscales (physical functioning, bodily pain, role limitations due to physical functioning, role limitations due to emotional problems, emotional well-being, social functioning, energy/fatigue, and general health perceptions) [22,23]. Each subscale represents a combined score (range: 0-100) derived from questions related to a particular health concept. The SF-36 has been shown to be both valid and reliable [22]. Mental component scale (MCS) and physical component scale (PCS) summary scores account for 81.5% of the reliable variance in the eight SF-36 scales and are therefore useful in providing a summarized interpretation of QOL [24,25]. In addition, these two scales have been scored in reference to a normal population (the 1998 general U.S. population, standard form) [26].

All participants who had previously signed an informed consent as part of their recruitment for the parent study gave a verbal informed consent prior to the conduct of each telephone interview. The study was approved by the Institutional Review Boards at the University of Southern California and the City of Hope.

### Statistical analysis

We compared demographic characteristics between herbal remedy users and non-users using Pearson chi-square tests for differences in frequency distributions.

Survival time was calculated as the time from the collection of herbal use at baseline telephone interview to the first of the following events: date of death or the end of follow-up on December 31, 2007. For breast cancer specific survival, we censored women who died from other causes on their dates of death. We used the Kaplan-Meier method to calculate survival rates and applied the log-rank test to test for any difference in survival between herbal remedy users and non-users.

Multivariable Cox proportional hazards regression models were fit separately for all-cause and breast cancer mortality to assess the hazard rate ratios of mortality associated with a woman's herbal remedy use within the 6 months prior to the baseline telephone interview. Hazard rate ratios, presented as relative risks (RRs) with 95% confidence intervals (CIs) were estimated using age in days at the start and end of follow-up to define a woman's participation time in the study.

Among women who participated in the follow-up telephone interview, we used t-tests to compare the QOL summary scale measures between herbal remedy users and non-users at baseline and follow-up surveys, respectively. We further applied multivariable linear regression models to compare average SF-36 health summary scale change scores (change score = follow-up score - baseline score) between herbal remedy users and non-users.

All multivariable models adjusted for the following factors, selected a priori, as potential confounders: ethnicity (non-Hispanic origin white, Hispanic origin white), age at diagnosis (< 35, 35-38, 39-40 years), stage of cancer at diagnosis (*in situ*, localized invasive, non-localized invasive), type of non-surgical treatment for initial breast cancer (no treatment, only chemotherapy, only radiation therapy, only hormonal therapy, any combinations of these therapies), type of surgery (lumpectomy, mastectomy), post-diagnosis cancer-related conditions (none, 1, ≥ 2 cancer conditions that included lymphedema, contralateral breast cancer, recurrence of the primary breast cancer, diagnosis of cancer at another site, and history of any breast cancer metastasis), comorbidities within six months of baseline interview (none, 1, ≥ 2 including cardiovascular diseases [defined as high blood pressure requiring medication, angina, having had an angiogram, high cholesterol, clotting disorder, or stroke], respiratory problems [asthma or allergy disorder affecting breathing], inflammatory conditions [arthritis, gall bladder disease, diabetes], musculoskeletal conditions [osteoporosis, recent fracture], nervous system disorders [migraines, hearing loss, psychiatric problem], drug abuse [excessive use of alcohol or problem with prescription or street drug use or dependency]), interval between diagnosis and initial interview (10-11, 12-13, 14-16 years), and annual income ($30,000, $30,001-60,000, $60,001-100,000, > $100,000) at baseline interview. In addition, when comparing average SF-36 health summary scale change scores between herbal remedy users and non-users, multivariable linear regression models were adjusted for the SF-36 scores at baseline.

Our analysis of mortality had limited statistical power. In estimating what we could expect to observe with 44 deaths during follow-up and a prevalence of 58.5% of participants who had used herbal remedies, we could detect a 60% or greater decrease in risk (RR = 0.4) or a 180% or greater increase in risk (RR = 2.8) for overall mortality with 80% statistical power given a 2-sided hypothesis test with a 5% level of statistical significance [27].

Two-sided *P*-values of 0.05 or less were considered statistically significant. We did not adjust *P *values for multiple comparisons as these analyses were considered as exploratory [28]. All analyses were performed using SAS version 9.2 (SAS Institute, Cary, NC).

## Results

### Demographic characteristics

Of 371 breast cancer patients, 217 (58.5%) reported having used herbal remedies within six months of baseline interview (Table 1). The most commonly used types of herbal remedies were echinacea (n = 107, 49%), herbal teas as a remedy (n = 77, 35%), and ginko biloba (n = 70, 32%), which together accounted for 77% (n = 166) of herbal remedy users. Compared to the non-users of herbal remedies, the users were more likely to have comorbidities and were less likely to have high income, but were similar to non-users on other factors (Table 2).

**Table 1:** Herbal remedy use^a ^among 371 participants

	Number of participants (%)
**Used herbal remedy(ies)**	217 (58.5)
**Among herbal remedy users**	
Echinacea	107 (49.3)
Herbal tea used as a remedy	77 (35.5)
Ginko biloba	70 (32.3)
Ginseng	48 (22.1)
St. John's Wort	47 (21.7)
Garlic	39 (18.0)
Evening primrose oil	26 (12.0)
Dong quai (tong Kwai)	18 (8.3)
Black cohosh	16 (7.4)
Bee Pollen	15 (6.9)
Progesterone topical cream (wild Mexican yam)	15 (6.9)
Valeriana	13 (6.0)
Wild yam root	10 (4.6)
Shark cartilage	9 (4.2)
Licorice root	9 (4.2)
Royal jelly	6 (2.8)
Chaste berries (vitex agnus cactii)	3 (1.4)
Chickweed tincture	2 (0.9)
Sage tea	3 (1.4)
Mother wort	1 (0.5)
Pulsatilla	1 (0.5)
Sepia	1 (0.5)
Sarsaparilla	0
Blue cohosh	0
False unicorn	0
Lachesis	0
Nux vomica	0
Others	35 (16.1)

^a^within the past six months of the baseline telephone survey

**Table 2:** Demographic characteristics of 371 participants

	Herbal remedy use		
	**No** **(n = 154)**	**Yes** **(n = 217)**	***P *value**^**a**^

Ethnicity			*0.28*
White	136 (40.6%)	199 (59.4%)	
Hispanic/Latino	18 (50.0%)	18 (50.0%)	
Age at diagnosis, years			*0.40*
< 35	37 (35.9%)	66 (64.1%)	
35-38	69 (43.4%)	90 (56.6%)	
39-40	48 (44.0%)	61 (56.0%)	
Stage of cancer at diagnosis			*0.14*
*In situ*	22 (43.1%)	29 (56.9%)	
Invasive local	94 (45.2%)	114 (54.8%)	
Invasive non-local	38 (33.9%)	74 (66.1%)	
Type of no-surgical treatment			
No treatment	40 (45.5%)	48 (54.5%)	*0.83*
Chemotherapy only	26 (41.9%)	36 (58.1%)	
Radiation therapy only	26 (44.1%)	33 (55.9%)	
Hormonal therapy only	5 (35.7%)	9 (64.3%)	
Any combination of therapies	57 (38.5%)	91 (61.5%)	
Type of surgery			*0.07*
Lumpectomy	39 (34.5%)	74 (65.5%)	
Mastectomy	115 (44.6%)	143 (55.4%)	
Post-diagnosis cancer-related conditions^b^			*0.26*
None	98 (44.1%)	124 (55.9%)	
1 cancer condition	35 (34.7%)	66 (65.3%)	
≥ 2 cancer conditions	21 (43.8%)	27 (56.2%)	
Comorbidities^c^			
None	39 (62.9%)	23 (37.1%)	*0.0005*
1 medication	47 (40.9%)	68 (59.1%)	
≥ 2 medications	68 (35.1%)	126 (64.9%)	
Interval between diagnosis and baseline interview, years			*0.62*
10-11	42 (37.8%)	69 (62.2%)	
12-13	55 (42.3%)	75 (57.7%)	
≥ 14	57 (43.9%)	73 (56.1%)	
Yearly income at baseline			*0.02*
≤ $30,000	18 (41.9%)	25 (58.1%)	
$30,001-$60,000	39 (38.2%)	63 (61.8%)	
$60,001-$100,000	32 (32.0%)	68 (68.0%)	
> $100,000	65 (51.6%)	61 (48.4%)	

*^a^P *value ascertained from Pearson's chi-square test.

^b^Post-diagnosis cancer-related conditions: breast cancer recurrence or metastasis, contralateral breast cancer, cancer at another site, lymphedema.

^c^Comorbidities: cardiovascular diseases, respiratory problems, inflammatory conditions, musculoskeletal conditions, nervous system disorders, drug abuse.

### Herbal remedy use and survival

**Figure 2:**
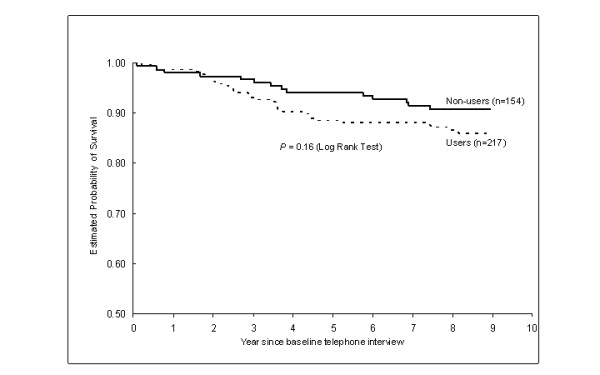
**Overall survival by herbal remedy use**.

Participants in the cohort were followed for an average of 7.8 years following their baseline telephone interview. During the follow-up, 44 women died, including 33 who died from breast cancer. The Kaplan-Meier survival curve showed that both overall survival and breast cancer survival of CAM users appeared to be poorer than that of non-CAM users, but the differences were not statistically significant (overall survival: *P *= 0.16, Figure 2; breast cancer survival: *P *= 0.08, figure similar to that for overall survival and not shown).

Overall herbal remedy use appeared to be associated with increased risks for all-cause mortality (RR = 1.28, 95% CI = 0.62-2.64), and breast cancer mortality (RR = 1.78, 95% CI = 0.72-4.40) (Table 3). Further, a non-statistically significant increase in risk for breast cancer mortality was associated with echinacea use (RR = 1.85, 95% CI = 0.80-4.29) and herbal tea used as a remedy (RR = 1.90, 95% CI = 0.83-4.35), but was not associated with ginko biloba use (RR = 0.99, 95% CI = 0.38-2.60).

**Table 3:** Adjusted^a ^relative risk of mortality associated with herbal remedy use among 371 participants, 1999-2007

	All-cause mortality			Breast cancer mortality		
	**No. Subjects**	**No. deaths**	**Adjusted**^**a **^**RR (95% CI)**	**No. Subjects**	**No. deaths**	**Adjusted**^**a **^**RR (95% CI)**

**Overall herbal remedy use**						
No	154	14	1.00	154	9	1.00
Yes	217	30	1.28 (0.62-2.64)	217	24	1.78 (0.72-4.40)
**By type**						
Echinacea use						
No	264	30	1.00	264	20	1.00
Yes	107	14	1.14 (0.55-2.35)	107	13	1.85 (0.80-4.29)
Herbal tea used as a remedy						
No	294	31	1.00	294	22	1.00
Yes	77	13	1.61 (0.78-3.29)	66	11	1.90 (0.83-4.35)
Ginko biloba use						
No	301	37	1.00	301	27	1.00
Yes	70	7	0.80 (0.34-1.89)	70	6	0.99 (0.38-2.60)

^a^Adjusted for ethnicity, age at diagnosis, stage of cancer at diagnosis, type of non-surgical treatment, type of surgery, post-diagnosis cancer-related conditions, comorbidities, interval between diagnosis and baseline interview, annual income at baseline interview.

### Herbal remedy use and QOL

Univariate analyses showed that at the baseline interview assessment, women reporting herbal remedy use had a lower mean SF-36 mental component summary score (*P *= 0.05) than women who reported no herbal remedy use, but their mean SF-36 physical component scale summary score was not statistically different from that of non-users (*P *= 0.37). However, 2-5 years after herbal remedy use information was collected, herbal remedy users' SF-36 mental and physical component summary scores were lower than those for non-users (all *P *< 0.05, results not shown).

Multivariable analyses of change scores between baseline and follow-up survey showed that both herbal remedy users' and non-users' mental component summary scores on the SF-36 increased similarly from baseline to follow-up (1.5 vs. 3.2, *P *= 0.16), but herbal remedy users' physical component summary scores decreased more than those of non-users (-5.7 vs. -3.2, *P *= 0.02, Table 4). Furthermore, herbal remedy users had a greater decline in the energy/fatigue (-7.3 vs. -1.8 vs., *P *= 0.02), physical role limitations (-13.7 vs. -3.9, *P *= 0.02), bodily pain (-11.4 vs. -3.4, *P *= 0.003), and general health (-6.4 vs. -2.1, *P *= 0.04) subscales and a smaller increase in the social functioning subscale (3.5 vs. 9.8, *P *= 0.04). No statistically significant difference in change scores was observed for the most commonly used specific types of herbal remedies (all *P *> 0.05).

**Table 4:** Adjusted^a ^mean differences^b ^in SF-36 summary and subscale scores between two interviews among 299 participants

	Overall herbal remedy use			Echinacea use			Herbal teas			Ginko biloba use		
	**No. (n = 120)**	**Yes (n = 179)**	***P *value**	**No.** **(n = 210)**	**Yes** **(n = 89)**	***P *value**	**No** **(n = 239)**	**Yes** **(n = 60)**	***P *value**	**No** **(n = 236)**	**Yes** **(n = 63)**	***P *value**

**Summary scale**												
PCS (physical health)	-3.2	-5.7	*0.02*	-4.6	-5.4	*0.46*	-4.6	-5.5	*0.47*	-5.0	-4.4	*0.64*
MCS (mental health)	3.2	1.5	*0.16*	1.9	2.7	*0.54*	2.4	1.4	*0.49*	1.8	2.9	*0.45*
**Subscale**												
Energy/fatigue	-1.8	-7.3	*0.02*	-5.2	-5.6	*0.87*	-5.1	-5.8	*0.82*	-5.1	-5.9	*0.79*
Physical functioning	-9.3	-11.8	*0.21*	-10.6	-11.4	*0.71*	-11.2	-9.9	*0.62*	-11.3	-9.7	*0.50*
Role limitations, physical	-3.9	-13.7	*0.02*	-10.1	-9.8	*0.96*	-8.5	-14.1	*0.30*	-11.0	-7.3	*0.47*
Emotional well-being	0.5	-0.5	*0.58*	-0.5	1.0	*0.45*	0.2	-1.2	*0.54*	-0.7	1.5	*0.32*
Role limitations, emotional	4.7	-1.6	*0.10*	0.7	0.7	*1.0*	1.2	-0.6	*0.70*	-0.7	4.7	*0.23*
Social functioning	9.8	3.5	*0.04*	5.3	7.2	*0.56*	7.8	0.7	*0.06*	5.2	7.4	*0.54*
Bodily pain	-3.4	-11.4	*0.003*	-7.5	-11.2	*0.19*	-7.3	-11.4	*0.21*	-9.0	-6.8	*0.49*
General health	-2.1	-6.4	*0.04*	-4.5	-5.8	*0.54*	-3.6	-7.7	*0.12*	-5.4	-2.9	*0.29*

**^a^**Adjusted for ethnicity, age at diagnosis, stage of cancer at diagnosis, type of non-surgical treatments, type of surgery, post-diagnosis cancer-related conditions, comorbidities, interval between diagnosis and first interview, yearly income at baseline interview, and the corresponding QOL measures at baseline interview. ^b^Difference = QOL measure at follow-up - QOL measure at baseline interview.

## Discussion

Herbal remedy use was associated with non-statistically significant increases in the risks for all-cause and breast cancer mortality. Both herbal remedy users' and non-users' mental component summary scores on the SF-36 increased similarly from the first survey to the second survey, but herbal remedy users' physical component summary scores decreased more than those of non-users.

This study has several important limitations which may affect the results we observed. First, although our results were obtained from a prospective study of young breast cancer patients who had survived more than 10 years, 276 (37%) breast cancer patients had died by the time we initiated this study, so that participants were more likely to be a select group of healthy women who were long-term survivors. Thus, our findings may not be generalizable to all breast cancer patients, but they do provide important information regarding long-term survivors of breast cancer who were diagnosed at a young age. Second, herbal remedy use, as measured in our study, represents use during a six month time period beginning, on average, 12.5 years after breast cancer diagnosis. We did not collect information regarding the timing of initiation of herbal remedy use, duration or frequency of use or reasons for use. Herbal teas were commonly used herbal remedies among our study population, but we did not collect the components of herbal teas or brand of herbal teas or whether any additives had been added to herbal teas to alter color or taste. Third, we relied on self reported information on herbal remedy use. Errors in reporting use of herbal preparations are possible. However, we worded questions carefully, mailed scales and questionnaire items in advance of the telephone interview to enhance comprehension and consistency of reporting, and limited the questions to recent herbal remedy usage (within the past six months of interview). Fourth, among 33 women who died from breast cancer during our follow-up for survival, 27 had post-diagnosis cancer-related conditions including two who had a contralateral breast cancer by the time of our baseline telephone interview. Unfortunately, we were unable to collect this information throughout the follow-up for survival. Therefore, we cannot rule out the possibility that women might have died of a new breast cancer rather than their first primary breast cancer. Fifth, due to the relatively small number of participants, we only assessed the effects for overall herbal remedy use and three commonly used herbal remedies. We were unable to explore the effects for those having a lower frequency of use among our participants. In fact, even for overall herbal remedy use, the small number of deaths led to insufficient statistical power to detect small or moderate differences in mortality risk between herbal remedy users and non-users. Due to the same limitation, our main exposure variable, herbal remedy use, grouped all types of herbal remedies. The definition of herbal remedy use was therefore non-specific. Finally, six of forty *P *values we reported here for testing the roles of herbal remedies in quality of life reached statistical significance (*P *≤ 0.05); we did not adjust for multiple comparisons as, with the relatively small number of participants, these analyses were consider as exploratory.

Our data show that herbal remedy use tended to be associated with a poor survival among long-term breast cancer survivors although the associations did not reach statistical significance, due to the small number of deaths and further deaths could alter the direction of findings. Our findings are consistent with a Norwegian study which followed 515 patients with a variety of cancers for up to 8 years and showed that CAM use was associated with poorer survival; 79% of CAM users died during follow-up compared with 65% of nonusers [10]. However, a second Norwegian study which followed 252 patients with heterogeneous cancers for up to 5 years did not find any association between CAM use and mortality [9].

Our data also showed that herbal remedy users' physical component summary scores decreased more than those of non-users between the first and second surveys. Most previous studies that have evaluated CAM or herbal remedy use in relation to QOL were conducted among patients within 5 years of initial diagnosis [11-14]; however, one study evaluated CAM use in a large sample of women who were diagnosed from 5 to 10 years earlier [16] and our prior report for this study population assessed the association between CAM use and QOL concurrently among 10-16 year survivors [20]. Among these studies, one found that CAM users (either all CAM or herbal CAM) had poorer physical and mental health function than non-users [11]; three showed poorer mental health function among CAM users compared to non-users [12,16,20], but not physical health function. A study conducted among Chinese breast cancer survivors found no association between any Chinese herbal medication use and perceived overall QOL [13]. However, in all but one study [12], the information on health-related QOL was collected at the same point in time as CAM use was collected. The study [12], which assessed QOL after CAM use, was able to determine if CAM use had been initiated prior to breast cancer treatment or subsequent to it, but covered only the 12 month period following initial diagnosis.

Reasons are unclear for our observation that herbal remedy use was associated with a poorer survival or greater decline in physical health summary scores between our two surveys. A recent published Norwegian study investigating CAM use in 735 cancer patients who had survived 5 years or more after breast cancer diagnosis, found CAM use initiated after diagnosis was associated with a poor prognosis suggesting that CAM might be used by those with less hope of a cure by conventional therapy [29]. Although no published data directly indicated that CAM use is associated with a lower score of QOL measures in the general population, previous data have shown that substantial amount of CAM use can be attributed to poorer health or the treatment of existing chronic health conditions [1,30-32]. Based on a national telephone interview survey conducted in the United States in 1997, 42% of all alternative therapies were used exclusively to treat existing illness [1]. Among breast cancer patients in one study, a variety of symptoms (treatment side effects as well as other health and cancer-related problems) have been positively related to the initiation of CAM use [12]. We found that herbal remedy users in our study were more likely to have comorbidities at the baseline interview assessment. The poorer survival and greater decrease in physical component summary scores among herbal remedy users may have resulted from poorer health motivating the use of remedies to alleviate symptoms although we controlled for comorbidities when comparing herbal remedy users and non-users. The differences in both survival and in the SF-36 physical component score between herbal remedy users and non-users remained when analyses were restricted to women without any comorbidities considered in our study.

We also considered that the lower physical QOL scores of the herbal remedy users might result from some of the users opting out of conventional therapy for breast cancer. Therefore, we adjusted for types of initial treatments (both surgical and non-surgical) in our analyses. Based on previous studies, approximately 5-12% of cancer patients may abandon appropriate therapy and pursue alternative treatments [33,34]; yet in most cases, alternative medicine is used to complement rather than replace conventional therapy [12,33,34].

It is possible that herbal remedy use could have interfered with chemotherapy treatment by inhibiting some enzymes in the cytochrome P450 (CYP) family, such as CYP1A2, CYP2C9, CYP2D6, CYP2E1, and CYP3A4 [35,36] if this use was initiated soon after diagnosis. This might explain the poorer survival and greater decline in physical health summary scores among herbal remedy users. However, as our participants were, on average, 13-year survivors, any potential interaction between chemotherapy treatment and concurrent herbal remedy use would not be expected to persist. Therefore, the associations that we observed in the current data need to be verified in the future studies with a larger sample size.

## Conclusions

Our data provide some evidence that herbal remedy use is associated with a poorer survival and a poorer physical component score for health-related QOL among long-term (> 10 years) breast cancer survivors. These conclusions are based on the exploratory data from a prospective study using two-sided statistical tests with no correction for multiple testing and are limited by the few deaths for the survival assessment and the lack of information regarding the timing of initiation of herbal remedy use or duration or frequency of, or reasons for herbal remedy use. The potential roles for herbal remedies in breast cancer patients' survival and QOL merit further exploration.

## Abbreviations

CAM: complementary and alternative medicine; CI: confidence interval; LA-CSP: Los Angeles County Cancer Surveillance Program; QOL: quality of life; RR: relative risk; SEER: Surveillance, Epidemiology, and End Results; SF-36: Short Form-36.

## Competing interests

The authors declare that they have no competing interests.

## Authors' contributions

HM obtained funding for the study of herbal remedies, carried out the statistical analysis and drafted the manuscript. LB obtained funding for the original study and the follow-up interviews, designed and implemented the original and follow-up studies, and supervised data collection, data management, and data cleaning. CLC assisted with questionnaire design and participated with JSH in data collection, data management, and data cleaning. All authors participated in the revision of the manuscript and have read and approved the final version.

## Pre-publication history

The pre-publication history for this paper can be accessed here:

http://www.biomedcentral.com/1471-2407/11/222/prepub
